# The Healthspan Project: A Retrospective Pilot of Biomarkers and Biometric Outcomes after a 6-Month Multi-Modal Wellness Intervention

**DOI:** 10.3390/healthcare12060676

**Published:** 2024-03-18

**Authors:** Elizabeth Chun, Annie Crete, Christopher Neal, Richard Joseph, Rachele Pojednic

**Affiliations:** 1 Department of Statistics, Texas A&M University, College Station, TX 77843, USA; 2 Restore Hyper Wellness, Austin, TX 78704, USArpojedni@norwich.edu (R.P.); 3 Department of Health and Human Performance, Norwich University, Northfield, VT 05663, USA

**Keywords:** whole-body cryotherapy, infrared sauna, photobiomodulation, compression, wellness, body composition, heart rate variability

## Abstract

Wellness-centric proactive healthcare is increasingly sought after, with individuals frequently embracing complementary modalities to achieve this goal. In this six-month study, healthy adult participants (n = 25) received specific therapies, including whole-body cryotherapy, infrared sauna, and photobiomodulation, along with guidance on physical activity, diet, and alcohol intake. Serum biomarkers were measured for all participants, while a subset also received biometric assessments for body composition (n = 10) and heart rate variability (n = 7). Over the course of the study (mean (SD) follow-up days = 174 (130)), participants exhibited significant improvements in health. LDL cholesterol (−9.77 (15.43) md/dL) and hsCRP (−1.75 (2.66) mg/L) decreased significantly (*p* < 0.05). HbA1c increased slightly (*p* < 0.05), but the effect size was small (0.12 (0.13)%). The body composition subset lost overall body weight (−3.29 (3.75) kg), primarily body fat, while preserving lean muscle mass (*p* < 0.05). Heart rate variability increased for those with existing cardiovascular risk factors (*p* < 0.05). In conclusion, participation in the multimodal Healthspan protocol is associated with substantial improvements in health-related biomarkers and biometrics.

## 1. Introduction

The wellness industry has surged in recent years, with the wellness economy representing 5.2% of the global impact in 2022 and market projections exceeding USD 8.7 trillion by 2027 [1]. This interest in wellness and proactive care can be explained in part by an upward shift in life expectancy [2], which has spawned a concomitant increase in the age-related disease burden, including cancer, heart disease, diabetes, and arthritis [3]. Thus, the burdens of an aging population have brought to the forefront a desire for preventive wellness interventions that reduce the strain of chronic morbidities. This concept has been more succinctly described as closing the gap between lifespan, the total years of one’s life, and healthspan, the years spent disease-free [4].

Traditional medicine has focused on ad hoc treatment as disease symptoms arise [5,6,7]. Such a disease-centric model of healthcare is undoubtedly effective in treating acute and infectious diseases. However, as chronic morbidities and aging-related conditions eclipse the prevalence of acute illness, this disease-centric model fails to address the changing landscape of medical care. Healthcare needs have shifted from acute diseases with simple, one-to-one etiologies toward chronic conditions with complex, multifaceted etiologies, a complexity that is poorly addressed by traditional medicine [5]. A new wellness-centric and proactive model of healthcare is needed to focus on comprehensive wellbeing that helps people expand their healthspans and live disease-free.

As the concept of healthy aging gains public interest, the need for scientific research is pressing. In particular, there is great interest in identifying interventions or protocols to improve proactive health and lengthen one’s healthspan [7], and health-conscious individuals are turning to a wide array of lifestyle interventions and complementary therapies [8]. Many such therapies, though perhaps replete with anecdotal evidence, lack a robust basis in the scientific literature. Indeed, interventions to treat comprehensive wellbeing and healthspan are under-researched, leaving crucial gaps in the scientific literature. 

Additionally, as part of the drive to bring a wellness-centric approach to healthcare, there is great need to bring together synergistic combinations of therapies that can adequately address the multifaceted etiology of modern healthcare challenges. Given the interconnectedness of many chronic diseases, as well as the prevalence of comorbidities, the future of medicine needs to involve a multi-modal model of healthcare.

This present study aims to analyze a multi-modal pilot intervention, the Healthspan Project, and its impacts on measures of overall wellness including standard blood-based biomarkers, body composition metrics, and cardiovascular fitness. It was hypothesized that participants would improve in all three categories with a dose-dependent effect of adherence.

## 2. Materials and Methods

### 2.1. Participants

Healthy adults over the age of 18 years participated in this study. Participants completed a health history intake form to screen for any contraindications or exclusion criteria. Participants were excluded if they reported a diagnosed chronic disease or untreated high/low blood pressure. Participants were also excluded if they were pregnant or breastfeeding. After the screening, all participants completed an informed consent form and waiver. Data were examined retrospectively and determined to be exempt by the Norwich University (Northfield, VT, USA) Institutional Review Board and Research Ethics Committee (HHS IORG #0004914, IRB #00005859).

### 2.2. Healthspan Therapies

Participants were enrolled in a 6-month multi-modal protocol at a commercial location. The multi-modal aspect indicates that participants engaged in more than one therapy as part of the protocol, which included simultaneous weekly treatments of whole-body cryotherapy (3 min per session, Zimno Tech, Wrocław, Dolnośląskie, Poland), infrared sauna (60 min per session, Sunlighten, Overland Park, KS, USA), photobiomodulation (11 min per session, PlatinumLED, Kailua, HI, USA), compression (30–60 min per session, Hyperice, Irvine, CA, USA), mild hyperbaric oxygen therapy (60 min per session, OxyHealth, Santa Fe Springs, CA, USA), and intravenous (IV) and intramuscular (IM) micronutrient therapy (Empower Pharmacy, Houston, TX, USA); they were also asked to participate in moderate exercise. The IV micronutrients included personalized combinations of vitamin C (500 mg), B vitamins (100 mg of Thiamine HCl, 2 mg of Riboflavin, 100 mg of Niacinamide, 2 mg of Dexpanthenol, and 2 mg of Pyridoxine HCl), and glutathione (400 mg), and the IM micronutrients included vitamin D (100,000 IUs), arginine (100 mg), ornithine (50 mg), lysine (50 mg), citrulline (50 mg), and a combination of methionine, choline, and inositol (2 mL), and B12 (1000 mcg). A summary of the intervention showing the average number of therapy sessions received per participant is reported in Table 1. Note that while all participants received therapies, only 16 have enumerated records for the amount of therapy received, which is summarized in the table. Participants self-reported engaging in regular physical activity, although this activity was not tracked during the study. 

### 2.3. Serum Biomarkers

Blood draws were conducted after an overnight fast at baseline and then incrementally at follow-up visits to track changes. The mean time between consecutive visits was 155 days (SD: 78 days), with the minimum being 17 and the maximum being 286 days. On average, there were 2.48 (SD 2.18) blood draw visits, with the total number of blood draws ranging from a minimum of 1 for the ten participants lost to follow-up to a maximum of 9 blood draws. For the 15 participants with at least one follow-up, there were on average 3.18 (SD: 2.35) total visits. The blood biomarkers that were measured consisted of metabolic analytes including fasting glucose, HbA1c, hsCRP, and serum lipids, including cholesterol (LDL and HDL) and triglycerides. All blood was drawn from the median cubital vein with a 20 gauge IV catheter needle. Drawn samples were centrifuged for 20 min at ambient temperature according to standard protocols (Drucker Diagnostics, Port Matilda, PA, USA) and sent to Quest Diagnostics (Secaucus, NJ, USA) for analysis. 

### 2.4. Body Composition

Body composition metrics were measured using an Inbody570 machine which calculates body composition from bioelectrical impedance (InBody USA, Cerritos, CA, USA). There were three overarching categories, fat mass, lean muscle mass, and water content, with individual data points including weight, body mass index (BMI), body fat mass (BFM), percent body fat, visceral fat level, arm circumference, and BFM and BFM% for left/right arms, left/right legs, and trunk. Raw impedance values were not analyzed, and the visceral fat level was re-encoded as a numeric value. 

### 2.5. Heart Rate Variability

Heart rate variability (HRV) data were recorded during sleep using an OURA ring (Oulu, Finland), which reports HRV using 5 min average intervals of the root mean square of successive differences (rMSSD) (ms) [9]. To calculate the rMSSD, successive differences between heart beats are squared and then averaged across regular intervals, in this case, 5 min. Then, the square root of the average squared difference is taken, yielding the final rMSSD value. Thus, the data consist of rMSSD measurements recorded every five minutes. The longest sleep segment per date was chosen to account for any naps that do not represent a true nighttime sleep segment. For participants who had HRV data formatted as arrays, the array values were unpacked into a flat dataframe for use in a downstream analysis. For participants with tabular HRV data, a sleep segment label was appended based on time gaps larger than 5 min to differentiate separate continuous sleep periods. Once the HRV data were pre-processed, they were summarized for each day of data collection by taking the overall average in the hour before waking of the longest sleep segment to follow the previous literature establishing morning as the optimal time for HRV measurement [10,11]. 

### 2.6. Statistical Analysis

Summary statistics, analyses, and statistical modeling were carried out in R v4.3.1 [12]. For data processing, the following packages were used from tidyverse v2.0.0: readr, readxl (file i/o), dplyr, hms, lubridate, tibble, stringr (wrangling), and ggplot2 (visualizations) [13]. 

Due to the small sample size, there was not enough power to separate individual therapy effects. Instead, overall participation in the protocol was studied by modeling time as the number of days since a participant began the program. For before and after comparisons, paired *t*-tests were conducted on the difference for all complete pairs of before and after data. Normality was tested using the Shapiro–Wilk test, and, where appropriate, the Wilcoxon signed-rank test was performed. Since test results, that is, the decision to accept or reject the null of no differences in means, were not different between the paired *t*-tests and the signed-rank tests, the *t*-test values were chosen. 

For the subset analyses, namely body composition and HRV, linear mixed-effects models were built using the nlme package v3.1-162 [14]. Mixed (random)-effects models were chosen due to repeated measures for each participant. 

For body composition metrics, each variable was first adjusted for participant-specific means and then modeled as a function of days from the start, age, sex, and height as the main fixed effects. All available metrics for fat mass, lean muscle mass, and water content, as described in Section 2.4, were modeled. In addition, biomarkers that were significantly different from start to end, namely LDL cholesterol, hsCRP, and HbA1c, were coded as binary levels (normal or elevated), with thresholds set as >100 mg/dL, >3.0 mg/L, and >5.7% for LDL, hsCRP, and HbA1c, respectively. These thresholds were chosen as they are above optimal ranges and indicate a higher health risk [15,16,17]. After inspection, HbA1c was always normal, so it was removed from consideration. The models were then fit with the main fixed effects as above, and hsCRP and LDL cholesterol levels were added as their main effects and interactions with days from the start. In all models, hsCRP and LDL were not significant after correcting for multiple comparisons, so they were removed. The final mixed-effects model for each variable consisted of days from start, age, sex, and height as fixed effects and participant as random. To correct for multiple testing, *p*-values were adjusted using the FDR method, and the threshold for significance was defined as an adjusted *p*-value (FDR) < 0.05.

For HRV, the data were first processed and averaged as described in the Heart Rate Variability section above. The average HRV was then modeled in a similar fashion as the body composition metrics. The response variable was the average HRV adjusted for participant-specific means, with the main effects being days from start, age, and sex. LDL cholesterol, hsCRP, and HbA1c levels were added with the same thresholds as for the body composition analysis, and HbA1c was subsequently removed as all participants had normal HbA1c values. In addition to the final overall model, one smaller model was fit to estimate the effect size for only those participants with both cardiovascular risk factors, i.e., elevated LDL cholesterol and hsCRP. The threshold for significance was set at *p* < 0.05.

## 3. Results

### 3.1. Participants

Altogether, there were 25 participants (12F/13M) in Healthspan, with ages on the study start date ranging from 31 to 64 years (mean (SD) years = 46.3 (10.1)). Participants were tracked for roughly six months (total follow-up days = 174 (130)). No participants reported any adverse events throughout the study, and there were no safety concerns reported by participants or clinical staff.

### 3.2. Biomarkers

A select number of biomarkers were recorded for all participants (n = 25) at the start of the study. At the end of the study, only 15 participants had biomarker data due to a loss of follow-up. Excluding those lost to follow-up, the minimum total time between the first and last blood draw was 34 days, and the maximum was 410 days. The mean values for all available participants at a given time point are given below in Table 2. Furthermore, these metabolic biomarkers were tested for the mean paired difference in before vs. after measurements. This comparison of the mean difference was carried out only with complete pairs, meaning participants who had both a before and an after measurement for that analyte. Fasting glucose, HDL cholesterol, and triglycerides were not significant at the *p* < 0.05 level. HbA1c increased slightly (0.12 (0.13)%, *p* < 0.05). Both hsCRP (−1.75 (2.66) mg/L) and LDL cholesterol (−9.77 (15.43) mg/dL) decreased at a significant level (*p* < 0.05). Note that while HbA1c did increase slightly, given the very small effect size and the fact that all participants maintained normal HbA1c levels, this result is not clinically relevant. In fact, differences of 0.5% or more are generally considered the threshold for clinically relevant changes in HbA1c, and the change observed here is below that threshold [18]. For a summary of blood-based biomarkers, see Table 2. 

### 3.3. Body Composition

There were ten (5F/5M) participants in the body composition subset, eight of whom had at least one follow-up measurement after their initial baseline. the average number of total body composition measurements was 5.60 (4.09), with the total follow-up time being approximately six months on average (179.9 (158.57) days). Intervals between consecutive visits ranged from the same day to 102 days between consecutive visits (mean (SD) days between visits = 40.94 (12.31). Note that the average age appears to drop slightly from start to end in Table 2. This is simply due to the loss of two participants to follow-up, thus causing a skew in the averages at study end.

A total of 36 body composition metrics were modeled as a function of days from Healthspan start. The sample size for each model was 56 observations across 10 participants. Of the 36 tested metrics, all fat-mass-related metrics showed a significant inverse association with days in Healthspan after correction with the FDR method (*p* < 0.05). In contrast, none of the lean-muscle-mass- or body-water-related metrics evidenced any significant association with days in Healthspan. Age and sex were also not significant in any models. Altogether, 16 metrics were significantly associated, and these metrics listed in order of significance are (smallest adjusted *p*-value first) weight, BFM, BMI, percent body fat, BFM right arm, BFM% right arm, BFM left arm, BFM% left arm, BFM trunk, BFM% trunk, BFM left leg, BFM% left leg, BFM% right leg, arm circumference, BFM right leg, and visceral fat level. Coefficient estimates of effect size for all significantly associated metrics were negative, indicating a decrease in all fat-mass-related measures over time. 

No significant difference was found in lean body mass metrics, which suggests that participants maintained lean muscle mass while specifically losing fat mass. When scaling the effect sizes to a standard six-month window, participants on average lost 2.2861 kg of body fat mass, with 1.0533 kg of loss in trunk fat mass specifically after adjusting for age, sex, and height. To see the 6-month scaled estimates for each metric, please see Table 3. 

**Figure 1:**
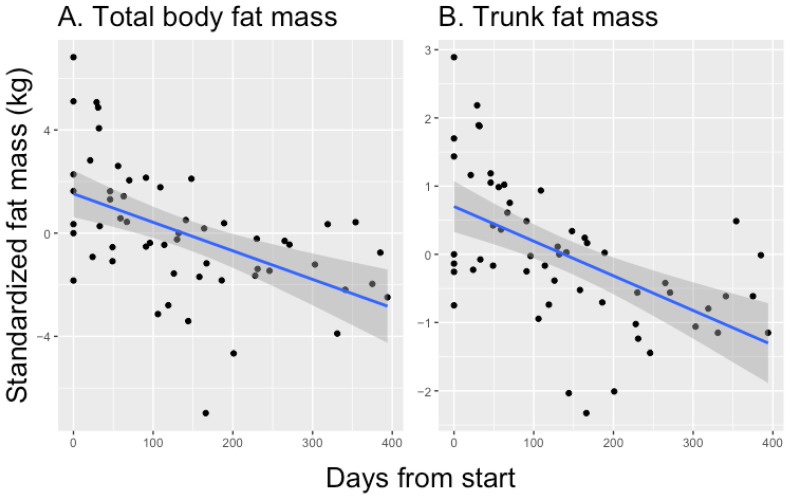
Selected body composition results: (**A**) total body fat mass and (**B**) target area (trunk) fat mass. The *y*-axis represents fat mass in kg, standardized by subtracting the participant-specific means, and the *x*-axis shows the number of days from the participant beginning the protocol. The black points represent the actual data. The blue lines show a smoothed average fit with the grey band representing a confidence region.

In addition to the summary of model estimates, a visualization of trends in body composition is of interest. To illustrate, two specific metrics of body fat, namely total body fat mass and the body fat mass of the trunk, are plotted in Figure 1. These two panels illustrate how body fat mass, both overall and in a target area, decreased over time with participation in the Healthspan protocol. 

### 3.4. Heart Rate Variability

There were seven participants (2F/5M) in the HRV subset. The minimum days of data collection was 20, with the average number of tracked days for all participants being 179.4 (120.59) days. 

**Figure 2:**
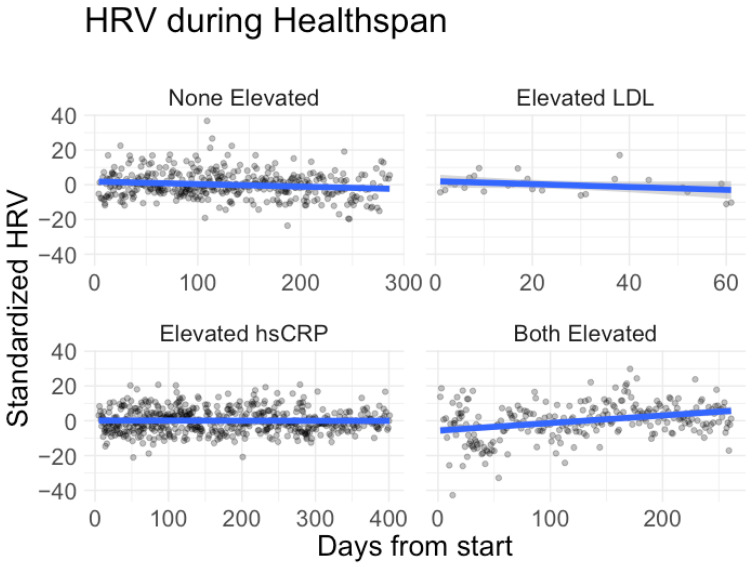
HRV results split by cardiovascular risk factors. HRV was standardized by subtracting the participant-specific means. The black points represent the actual data. The blue lines show a smoothed average fit with the grey band representing a confidence region.

In the overall model, the interaction effects for days from the start with elevated LDL and elevated hsCRP were positively associated with average HRV. This means for participants with a cardiovascular risk factor, taking part in Healthspan showed a statistically significant increase in HRV over time. Age and sex were not significant. When scaled to a standard 6-month window (180 days), the average HRV increased by 9.3273 ms for participants with both risk factors (*p* < 0.05). See Table 4 for more details on HRV model results. For a visualization of the overall trends by group from the start to the end of study, please see Figure 2. 

These results suggest that the Healthspan protocol may have particular benefit to those who already have some cardiovascular risk factors.

## 4. Discussion

Overall, participants in the Healthspan protocol saw significant improvements in their health across blood metabolic biomarkers. In addition, the body composition subset showed a benefit for fat mass loss, while the subset for HRV showed a benefit for those with cardiovascular risk factors.

In the biomarker analysis, participants had significantly decreased LDL cholesterol and hsCRP. Only HbA1c increased with a very small effect size. Although it is impossible to determine the exact cause of this small increase, it could be due to external factors such as diet and physical activity, which were not monitored. As for the lipid and hsCRP results, these decreases are particularly meaningful given the prevalence and long progression of cardiovascular diseases through the human lifespan [19]. Decreases in LDL cholesterol can help reduce major vascular events [20], and having low hsCRP, defined as <2 mg/L, can have protective effects against stroke and coronary heart disease [21]. The hsCRP results are particularly notable given that the participants in the present study saw their hsCRP decrease on average from above the 2 mg/L threshold at the start of the study to below this threshold at the end of the study. 

For body composition, participants saw beneficial effects, namely a loss of total body weight, total body fat, and body fat in each target area. The ability to study body composition on a granular and targeted level is crucial in assessing overall health impact. Indeed, while health risk is not necessarily correlated with overall weight, it is associated with body composition, especially visceral fat and abdominal adiposity [22,23]. Body composition analyses were complex and cost-prohibitive in the past [24,25], but the emergence of bioelectrical impedance analysis technology, such as the Inbody used in the present study, have enabled their wider-scale implementation [26]. The results from this study demonstrate the importance of body composition measurements by showing significant decreases in all metrics of body fat while showing no effect on lean muscle mass. In other words, participants lost not only weight but the specific components of weight that contribute to health risk while maintaining healthy muscle.

The HRV analysis also showed positive benefits to cardiovascular health over time for specific subsets. In particular, those with elevated LDL cholesterol, elevated hsCRP, or both saw their HRV increase on average. Generally, low HRV is associated with decreased health and increased mortality risk, while higher HRV often indicates better fitness and adaptability [27,28]. While there are several methods of measuring HRV, the metric studied here, namely rMSSD, is regarded as a reliable measure with low influence or error from respiratory effects [29]. Additionally, the study of HRV via wearables like the Oura ring is a new and emerging field of research with great potential. While the importance of HRV in monitoring health and fitness has long been established, research and applications in the field have been limited by the need to collect data via electrocardiogram [30]. The advent of wearable devices that can accurately track HRV enables the broad, public study of this important health metric [31]. The present study advances the field of HRV research by using wearables data to demonstrate a statistically significant improvement in HRV over the course of a real-world multi-modal wellness intervention. In addition to showing the statistical increase in HRV through Healthspan, it is also vital to consider the clinical relevance of changes in HRV. The smallest beneficial change in HRV as measured via rMSSD has been proposed to be +3% [32]. In this study, benefits for the three groups with cardiovascular risk factors showed increases of between 10 and 35% on average. Thus the effect of Healthspan on HRV was not only statistically significant but also evidenced a clinically meaningful change. 

As people shift towards a wellness-centric model that addresses whole-body health in concert, a model of combining therapies—both in practice and research—is becoming crucial. Through the multi-modal design of the Healthspan protocol, this study sought to improve not just a single aspect of health but rather to benefit overall wellness with a multi-pronged approach which mimics real-world practice. Each therapy in Healthspan has unique yet complementary effects. Cryotherapy is known to act on inflammation and may help with glucose metabolism [33,34]. Sauna therapy continues the anti-inflammatory focus and also adds beneficial impacts for cardiovascular health and lipid metabolism [35,36,37,38]. Photobiomodulation has been proposed to beneficially impact mitochondrial energy production, and it is also used to improve cognitive function, muscle recovery, and pain relief [39,40,41,42,43]. Muscle recovery and pain relief are further addressed by compression therapy, which helps to increase blood flow and reduce lymphedema [44,45,46,47]. The final component of the Healthspan protocol was micronutrient therapy in the form of both IV and IM supplementation. Micronutrient therapy can have a variety of effects based on the specific micronutrients administered, with benefits including, though not limited to, antioxidant protection, glucose homeostasis, amino acid synthesis, reduced hypertension, and improved cognitive function [48,49,50,51,52]. These micronutrients can work both alone and in conjunction with the other therapies in the Healthspan protocol, further enhancing the wellness-centric approach to health.

Altogether, these results suggest that the multi-modal Healthspan protocol is effective in improving overall health with analyses of blood biomarkers, body composition, and HRV. It is probable that the combination of therapies had a synergistic effect resulting in greater wellness gains than any of the therapies in isolation. For example, a study by de Brito et al. found the combination of cryotherapy and antioxidant vitamin therapy showed a greater impact on exercise recovery than either cryotherapy or vitamins alone [53]. In particular, an increase in antioxidant capacity was seen only when the therapies were combined, suggesting a synergistic effect of the association. As another example of synergistic effects, cryotherapy in combination with compression was found to be superior in pain relief for those recovering from anterior cruciate ligament surgery as compared to cryotherapy alone [54]. Photobiomodulation may also benefit from synergy with other modalities, in particular micronutrient therapy. A study of participants with Hashimoto thyroiditis found that the combination of photobiomodulation and specific micronutrients resulted in better thyroid function than supplementation alone [55]. 

Altogether, the therapy modalities in the Healthspan protocol each show the potential for benefits in the existing literature. The current study builds upon the underlying mechanistic studies to show that these therapies have a quantifiable real-world impact. A strength of this study is the fact that it was performed in a free-living, real-world environment in which participants incorporated the Healthspan protocol into their daily lives. One benefit is the immediate applicability of the results outside of clinical practice. In particular, no rigid treatment structure is required, and Healthspan is immediately applicable for the general public. 

However, such a design also has drawbacks. A distinct limitation is the lack of control over outside variables. Participants were free to go about their lives without adhering to a controlled environment, and participation in therapies was not entirely structured. Additionally, the follow-up time was not standardized. This could affect start-to-end comparisons since participants had different durations of therapy. For example, if an effect takes time to manifest, participants with a very short follow-up period might shrink the overall start-to-end difference toward zero, thus reducing the power to find true effects. In addition, although participants were instructed to perform moderate exercise, this activity was not tracked or monitored. Given the design, the specific effects of different therapies could not be separated. A related limitation is sample size, especially for the biometric data. Because of the small sample, the generalizability of results is uncertain. However, the benefit of this pilot study is in showing potential effects and providing a direction for future research. A future study could employ a larger sample size in order to focus on teasing apart therapy-specific effects. It is also possible that certain therapies are more effective in specific combinations. Further study is warranted, perhaps with a factorial design, to determine the optimal combinations and orderings of therapies.

## 5. Conclusions

In summary, the multimodal Healthspan intervention shows benefits for overall wellbeing in a proactive and preventive approach to healthcare. Each individual therapy component of the protocol has been previously studied in the literature for its specific effects on health. This study builds upon those known results to show improvements in overall health in a real-world, free-living environment. In particular, participants improved their blood-based biomarkers, namely LDL cholesterol and hsCRP, along with body composition, especially body fat mass, and lastly HRV for a subset of higher-risk participants. 

## Figures and Tables

**Table 1:** A summary of the intervention showing the number of therapy sessions received per participant. WBC = whole-body cryotherapy, PBM = photobiomodulation, also called red light therapy, mHbOT = mild hyperbaric oxygen therapy, IV = intravenous micronutrients, and IM = intramuscular injection. Note that this summary was calculated using participants with recorded amounts of therapy (n = 16), but all participants (n = 25) were involved in the protocol and received therapies.

Therapy	Summary Mean (SD)
WBC	46.88 (44.97)
IR Sauna	17.69 (19.96)
PBM	40.31 (49.30)
Compression	13.50 (16.80)
mHbOT	21.13 (18.92)
IV *	27.06 (16.47)
IM *	15.63 (10.54)
n	16
Days in intervention	213.88 (115.32)

* IV micronutrients included personalized combinations of vitamin C (500 mg), B vitamins (100 mg Thiamine HCl, 2 mg Riboflavin, 100 mg Niacinamide, 2 mg Dexpanthenol, 2 mg Pyridoxine HCl), glutathione (400 mg); IM micronutrients included vitamin D (100,000 IUs), arginine (100 mg), ornithine (50 mg), lysine (50 mg), citrulline (50 mg), and a combination of methionine, choline, and inositol (2 mL), and B12 (1000 mcg).

**Table 2:** Demographic information and biomarkers.

	Variable	Before Mean (SD)	After Mean (SD)	Paired Difference Mean (SD) ^a^	Paired Test *p*-Value ^b^
Healthspan Overall	n	25	15	NA	NA
	Sex	12F/13M	8F/7M	NA	NA
	Age (years)	46.3 (10.1)	49.8 (9.35)	NA	NA
	HbA1c (%)	5.10 (0.32)	5.29 (0.30)	0.12 (0.13)	0.012 *
	Fasting glucose (mg/dL)	96.91 (13.71)	98.00 (14.61)	−1.62 (15.62)	0.716
	hsCRP (mg/L)	2.87 (3.19)	1.79 (1.93)	−1.75 (2.66)	0.043 *
	LDL (mg/dL)	124.08 (51.13)	107.08 (38.86)	−9.77 (15.43)	0.041 *
	HDL (mg/dL)	58.24 (13.20)	57.31 (14.04)	−0.46 (7.56)	0.829
	Triglycerides (mg/dL)	131.24 (99.48)	110.46 (57.54)	−10.15 (43.49)	0.416
Body Composition Subset	n	10	8	NA	NA
	Sex	5F/5M	4F/4M	NA	NA
	Age (years)	44.7 (8.16)	42.9 (8.25)	NA	NA
	Weight (kg)	88.83 (14.33)	86.61 (14.75)	−3.29 (3.75)	0.042 *
HRV Subset	n	7	7	NA	NA
	Sex	2F/5M	2F/5M	NA	NA
	Age (years)	43.3 (7.8)	43.9 (7.7)	NA	NA

* denotes *p* < 0.05. ^a^ Mean and standard deviation (SD) values for paired differences (after–before) were calculated for complete pairs of before vs. after measurements. ^b^ A two-tailed *t*-test was used for differences between complete pairs.

**Table 3:** Selected body composition results: significantly associated metrics, excluding % metrics, in order of adjusted *p*-value (smallest first). Visceral fat level is categorized in levels from 1 to 20. Six-month scaled estimates are simply the model estimates multiplied by 180 days. Note the original data have been converted to metric units using the following formulas: 1 lb/2.2045 = x kg and 1 in/2.54 = x cm.

Metric	Model Estimate (units/day)	6-Month Scaled Estimate	Adjusted *p*-Value
Weight (kg)	−0.0127	−2.2861	0.0000
Body fat mass (BFM) (kg)	−0.0129	−2.3270	0.0000
Body mass index (BMI) (kg/m^2^)	−0.0050	−0.9000	0.0000
BFM right arm (kg)	−0.0018	−0.3184	0.0000
BFM left arm (kg)	−0.0018	−0.3184	0.0000
BFM trunk (kg)	−0.0059	−1.0533	0.0000
BFM left leg (kg)	−0.0016	−0.2939	0.0003
Arm circumference (cm)	−0.0043	−0.7772	0.0005
BFM right leg (kg)	−0.0015	−0.2694	0.0010
Visceral fat (levels)	−0.0042	−0.7560	0.0018

**Table 4:** HRV model results showing the estimated effect size in HRV (ms) per day for specific groups of interest. Elevated LDL and hsCRP were defined as >100 mg/dL and >3.0 mg/L, respectively. Six-month scaled estimates are simply the model estimates multiplied by 180 days, and the scaled % increase is the increase relative to the overall average HRV across participants.

Group	Model Estimate (ms/day)	6-Month Scaled Estimate (ms/day)	Scaled Estimate as % Increase	*p*-Value
Elevated LDL ^a^	0.0509	9.1665	35.00%	0.0000
Elevated hsCRP ^a^	0.0150	2.6970	10.30%	0.0088
Both elevated ^b^	0.0518	9.3273	35.61%	0.0000

^a^ Model estimates from full model with all participants. ^b^ Model estimates from reduced model with only participants having both risk factors elevated.

## Data Availability

The source data are available to verified researchers upon request by contacting the corresponding author.
